# Precise annotation of tick mitochondrial genomes reveals multiple copy number variation of short tandem repeats and one transposon-like element

**DOI:** 10.1186/s12864-020-06906-2

**Published:** 2020-07-17

**Authors:** Ze Chen, Yibo Xuan, Guangcai Liang, Xiaolong Yang, Zhijun Yu, Stephen C. Barker, Samuel Kelava, Wenjun Bu, Jingze Liu, Shan Gao

**Affiliations:** 1 Hebei Key Laboratory of Animal Physiology, Biochemistry and Molecular Biology, College of Life Sciences, Hebei Normal University, Shijiazhuang, Hebei 050024 P. R. China; 2 College of Life Sciences, Nankai University, Tianjin, Tianjin, 300071 P. R. China; 3 Frontier Science Center for Synthetic Biology and Key Laboratory of Systems Bioengineering (Ministry of Education), School of Chemical Engineering and Technology, Tianjin University, Tianjin Tianjin, 300350 P. R. China; 4 School of Chemistry and Molecular Biosciences, The University of Queensland, Brisbane, QLD 4072 Australia; 5 School of Statistics and Data Science, Nankai University, Tianjin Tianjin, 300071 P. R. China

**Keywords:** Mitochondrial DNA, Precise annotation, Short tandem repeat, Transposon, Tick

## Abstract

**Background:**

In the present study, we used long-PCR amplification coupled with Next-Generation Sequencing (NGS) to obtain complete mitochondrial (mt) genomes of individual ticks and unprecedently performed precise annotation of these mt genomes. We aimed to: (1) develop a simple, cost-effective and accurate method for the study of extremely high AT-content mt genomes within an individual animal (e.g. *Dermacentor silvarum*) containing miniscule DNA; (2) provide a high-quality reference genome for *D. silvarum* with precise annotation and also for future studies of other tick mt genomes; and (3) detect and analyze mt DNA variation within an individual tick.

**Results:**

These annotations were confirmed by the PacBio full-length transcriptome data to cover both entire strands of the mitochondrial genomes without any gaps or overlaps. Moreover, two new and important findings were reported for the first time, contributing fundamental knowledge to mt biology. The first was the discovery of a transposon-like element that may eventually reveal much about mechanisms of gene rearrangements in mt genomes. Another finding was that Copy Number Variation (CNV) of Short Tandem Repeats (STRs) account for mitochondrial sequence diversity (heterogeneity) within an individual tick, insect, mouse or human, whereas SNPs were not detected. The CNV of STRs in the protein-coding genes resulted in frameshift mutations in the proteins, which can cause deleterious effects. Mitochondria containing these deleterious STR mutations accumulate in cells and can produce deleterious proteins.

**Conclusions:**

We proposed that the accumulation of CNV of STRs in mitochondria may cause aging or diseases. Future tests of the CNV of STRs hypothesis help to ultimately reveal the genetic basis of mitochondrial DNA variation and its consequences (e.g., aging and diseases) in animals. Our study will lead to the reconsideration of the importance of STRs and a unified study of CNV of STRs with longer and shorter repeat units (particularly polynucleotides) in both nuclear and mt genomes.

## Background

Annotation of mitochondrial (mt) genomes is indispensable for fundamental research in many fields, including mt biochemistry, physiology, and the molecular phylogenetics and evolution of animals. Moreover, high-resolution annotation of animal mt genomes can be used to investigate RNA processing, maturation, degradation and even the regulation of gene expression [1]. In our previous studies, two substantial contributions to the methods used to annotate mt genomes have been published. The first one was that Gao et al. constructed the first quantitative transcription map of animal mt genomes by sequencing the full-length transcriptome of the insect *Erthesina fullo* Thunberg [1] on the PacBio platform [2]. Novel findings included the 3′ polyadenylation and possible 5′ m^7^G caps of rRNAs [1], the polycistronic transcripts [1], the antisense transcripts of all mt genes [1], and novel long non-coding RNAs (lncRNAs) [3]. Based on these findings, we proposed the uninterrupted transcription of mammal mt genomes [3]. In addition, we proposed that long antisense transcripts degrade quickly as transient RNAs, making them unlikely to perform specific functions [4], although all antisense transcripts are processed from two primary transcripts. The second contribution concerned the use 5′ and 3′ end small RNAs (5′ and 3′ sRNAs) [4] to annotate mt genes to a resolution of 1 bp, subsequently dubbed “precise annotation” [5]. Precise annotation of these accurate genomes led us to discover a novel 31-nt ncRNA in mammalian mt DNA [4] and that the copy numbers of tandem repeats exhibit great diversity within an *E. fullo* individual [5]. Recently, precise annotation of human, chimpanzee, rhesus macaque and mouse mt genomes has been performed to study five Conserved Sequence Blocks (CSBs) in the mt D-loop region [6]; this ultimately led to a deep understanding of the mechanisms involved in the RNA-DNA transition and even the functions of the D-loop.

In the present study, we used long-PCR amplification coupled with Next-Generation Sequencing (NGS) to obtain complete mt genomes of individual ticks and performed precise annotation of these mt genomes. Given that conventional mtDNA isolation and purification are not required in our method and in the Whole-Genome Sequencing (WGS) method, both the WGS method and our method are simple and cost-effective. However, compared to the WGS method, our method has three main advantages: (1) errors in the assembly of mt genomes caused by highly similar exogenous or nuclear sequences [i.e., Nuclear Mitochondrial DNA (NUMT)] are avoided; (2) highly similar segments (e.g., control regions 1 and 2 of *Dermacentor silvarum*) of mt genomes can be assembled separately (Results); and (3) sequence heterogeneity and DNA variation in mt genomes within an individual can be accurately determined due to the high depth of sequencing data. In the present study, we aimed to achieve the following research goals: (1) develop a simple, cost-effective and accurate method for the study of extremely high AT-content mt genomes within an individual animal (e.g. *D. silvarum*) containing miniscule DNA; (2) provide a high-quality reference genome for *D. silvarum* with precise annotation and also for future studies of other tick mt genomes; and (3) detect and analyze mt DNA variation within an individual tick.

## Results

### Using long-PCR and NGS to obtain complete mt genomes of individual ticks

**Fig. 1:**
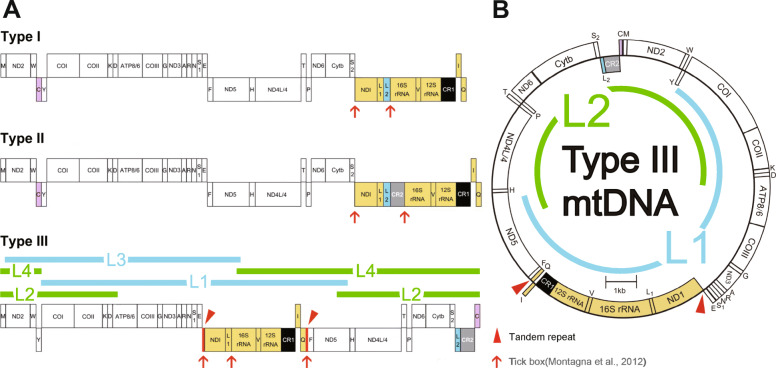
Long-PCR amplification of each entire mt genome. All the primers and PCR reaction conditions are listed in Table 1. The tRNA genes are represented by their single letter codes. CR1 and CR2 represents the control region 1 and the control region 2, respectively. Translocated genes are reported in the same colour. All the reference genomes of tick mitochondria read in the 5′ → 3′ direction as the major coding strand (J-strand). Genes on the J-strand and the N-strand are shown high and low, respectively. **a**. The tick mt genomes were classified into three types, which are type I, II and III (Results). The type III mt genomes were amplified into two large segments (L1&L2 or L3&L4) by long-PCR using total DNA from individual ticks. Using the complete *D. silvarum* mt genome (GenBank: MN347015), L1, L2, L3 and L4 were estimated as ~ 9.6, ~ 8.2, ~ 7.2 and ~ 9.2 Kbp in size (Table 1), respectively. **b**. The type III mt genomes of ticks read clockwise in the 5′ → 3′ direction

**Fig. 2:**
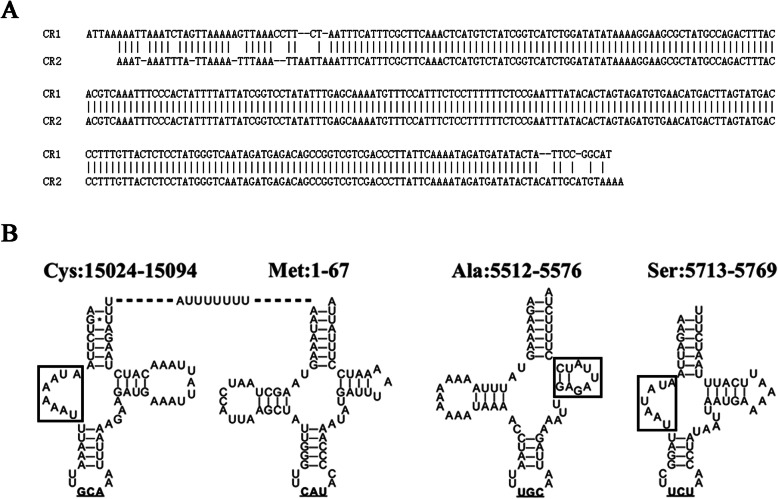
Precise annotation of mt tRNAs and control regions. **a**. CR1 and CR2 were determined in the *D. silvarum* mt genome (GenBank: MN347015). **b**. In MN347015, small RNA A[U]7 was produced from between tRNA^Cys^ and tRNA^Met^. One of tRNA^Ser^ and tRNA^Cys^ had no D-arms, whereas tRNA^Ala^, tRNA^Glu^, tRNA^Tyr^ and tRNA^Phe^ had unstable T-arms (indicated in black box)

**Fig. 3:**
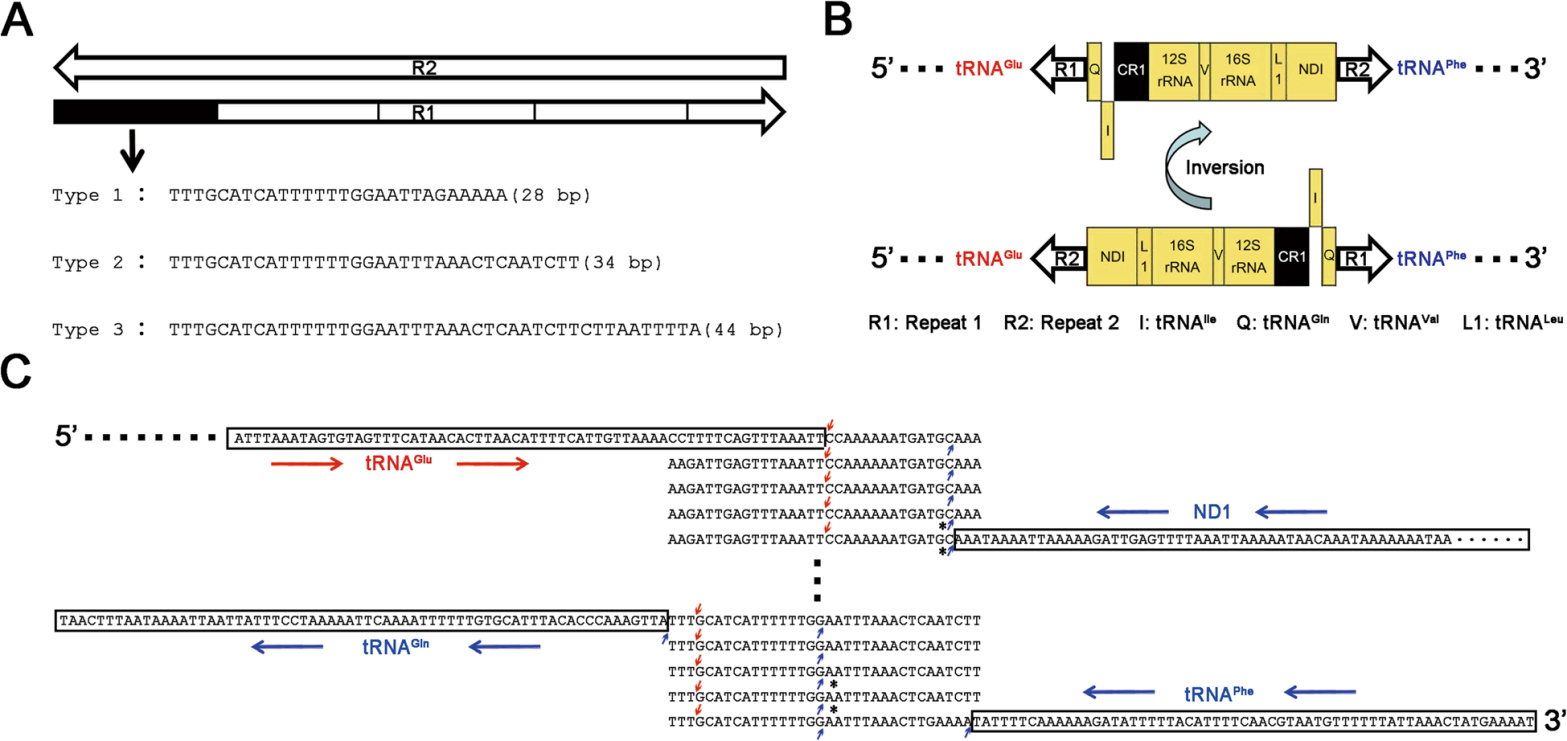
The transposon-like element in the *Dermacentor silvarum* mt genome. All the mt genomes read in the 5′ → 3′ direction as the J-strand. The genes from the J-strand and the N-strand are indicated in red and blue colours, respectively. **a**. The genes from the J-strand and the N-strand are deployed upward and downward, respectively. **b**. R1 and R2 were composed of several repeat units, respectively. And the repeat units in R1 are reverse complimentary to those in R2. In total, three types of repeat units (type 1, 2 and 3) of R1 were identified. **c**. R1 and R2 were determined to have 5 repeat units in the *D. silvarum* mt genome (GenBank: MN347015)

A previous study [7] classified tick mt genomes into three types according to gene orders (Fig. 1a): (1) type I for Argasidae (soft ticks) and non-Australasian Prostriata (“other *Ixodes*”); (2) type II for Australasian Prostriata (“Australasian *Ixodes*”); and (3) type III for Metastriata (all other hard ticks). The nomenclature “other *Ixodes*” and “Australasian *Ixodes*” is from [8]. The present study focused on the genus *Dermacentor* belonging to Metastriata using ticks from four species (*D. silvarum, D. nuttalli, D. marginatus* and *D. niveus*). The type III mt genomes of individual ticks (Fig. 1b) were obtained using long-PCR amplification coupled with NGS (Methods). All the reference genomes of tick mitochondria read in the 5′ → 3′ direction as the major coding strand (J-strand). Using specific primers (Table 1), each entire mt genome was amplified in two large segments: large segment 1 (L1) and large segment 2 (L2) or large segment 3 (L3) and large segment 4 (L4). L1 and L2 contain Control Region 1 (CR1) and Control Region 2 (CR2), respectively, whereas L3 and L4 contain tandem Repeat 1 (R1) and tandem Repeat 2 (R2), respectively (Fig. 1a). Using ~ 4 Gbp 2 × 150 DNA-seq data for each genome, the complete mt genomes of *D. silvarum*, *D. nuttalli* and *D. marginatus* were obtained by assembling L3 and L4 separately then merging L3 and L4 (Fig. 1b). In addition, CR1 and CR2 on L4 were validated using PCR amplification coupled with Sanger sequencing, separately, as CR1 and CR2 share an identical segment (Fig. 2a). Using ~ 4 Gbp 2 × 150 DNA-seq data, the complete mt genome of *D. silvarum* was also obtained by assembling L1 and L2 separately then merging L1 and L2. Furthermore, R1 and R2 on L1 were validated using PCR amplification coupled with Sanger sequencing, separately, as the repeat units of R1 are the reverse complements of the repeat units of R2 (Fig. 3). Comparison of the *D. silvarum* mt genomes obtained by sequencing L3 and L4 with those obtained by sequencing L1 and L2 improved the accuracy of the DNA sequence. Since both R1 and R2 were longer than 150 bp, we also used ~ 4 Gbp 2 × 250 bp DNA-seq data to obtain full-length sequences of R1 and R2 for genome polishing. In total, 12.7 Gbp DNA-seq data were generated to cover ~ 848,069 × (12.72 Gbp/1.5 Kbp) of the *D. silvarum* mt genome (GenBank: MN347015) which was used as a reference for precise annotation in the following studies.

**Table 1:** PCR primers for the *Dermacentor* mt genomes

Forward primer	Reverse primer	Segment	Size(bp)
TCAGTCATTTTACCGCGATGA	GCTCAAATTCCATTCTCTGC	L1	9580
AGCTGTTACTAACGTTGAGG	AGGATGTTGATGGATCGAAA	L2	8156
GCTAKTGGGTTCATACCCCAA	CGACCTCGATGTTGGATTAGGA	L3	7155
CCAACCTGATTCWCATCGGTCT	TCATCGCGGTAAAATGACTGA	L4	9187
TGCTGCTGGCACAAATTTAGC	CAAGATGACCCTAAATTCAGGCA	CR1	483
GGAGCTATACCAATTGAATATCCC	TTGGGGTATGAACCCAATAGC	CR2	645
TGCATTCAGTTTCGGCCTGA	CCGGCTGTCTCATCTATTGAC	R2	3616
CTATTCCGGCATAGTAAAATGCCTG	CAAGCTTATGCACCCTTTTCAATAC	R1	570

These primers were designed to amplify large segments (L1, L2, L3 and L4) and short segments (CR1, CR2, R1 and R2) in the mt genomes of the genus *Dermacentor*. Their PCR reaction conditions can be seen in the Methods. Based on the results using 100 individual ticks from four species, the primers for L3 and L4 were optimized to amplify more species of the genus *Dermacentor* than those of L1 and L2. The R2 segment spanned tRNA^Arg^, tRNA^Asn^, tRNA^Ser^, tRNA^Glu^, ND1, tRNA^Leu^, 16S rRNA, tRNA^Val^, 12S rRNA and CR1. The R1 segment spanned tRNA^Ile^, tRNA^Gln^, R1, tRNA^Phe^ and ND5. The segment sizes were estimated using the *D. silvarum* mt genome (GenBank: MN347015)

Comparison of *D. silvarum, D. nuttalli* and *D. marginatus* mt genomes showed that they have the same gene order (type III) and high sequence identities (> 95%) (tandem repeats were not part of these calculations). Preliminary analysis showed two significant features in these tick mt genomes that are also possible in other ticks of Metastriata (Fig. 1a): (1) the mt genomes of *D. silvarum, D. nuttalli and D. marginatus* contained two tandem repeats (R1 and R2); and (2) these tick mt genomes contained multiple Short Tandem Repeats (STRs) with very short repeat units (1 or 2 bp). STRs, widely used by forensic geneticists and in studies of genealogy, are often referred to as Simple Sequence Repeats (SSRs) by plant geneticists or microsatellites by oncologists. Found widely in animal mt genomes, STRs follow a pattern in which one or more nucleotides (repeat unit) are repeated and the repeat units are directly adjacent to each other, allowing for very rare Single Nucleotide Polymorphisms (SNPs) in the repeat units. The minimum length of the repeat units of STRs is obviously 1 bp; we call type of STR a polynucleotide. PolyAs and polyTs occur frequently in tick and insect mt genomes; indeed, they contribute substantially to the high AT content of many of these mt genomes. Polynucleotides and tandem repeats R1 and R2 had the same pattern of variation in the *D. silvarum* mt genome (**below**). This suggested that a unified study should be performed on the CNV of STRs with longer and shorter repeat units, particularly polynucleotides that were usually overlooked in previous studies. To describe a tandem repeat, we use the repeat unit and its copy number. STRs can be classified by their repeat unit length (m) and copy number (n), thus briefly noted as m × n STR. For example, the STR ATATATATAT is noted as [AT]_5_ and classified as 2 × 5 STR. In this way, a polynucleotide is classified as 1 × n STR.

### Precise annotation of the *Dermacentor silvarum* mt genome

Our *D. silvarum* mt genome shares a sequence identity of 97.47% with the publicly available *D. silvarum* mt genome NC_026552.1 in the NCBI RefSeq database. We performed precise annotation of the complete *D. silvarum* mt genome (Table 2) using sRNA-seq data and confirmed these annotations using the PacBio full-length transcriptome data (Methods). Although most of the new annotations were consistent with those of NC_026552.1, we corrected many errors in NC_026552.1, particularly in tRNAs, rRNAs, CR1, CR2, R1 and R2. *D. silvarum* transcribes both entire strands of its mt genome to produce primary transcripts covering CR1 and CR2, predicted to be non-coding and non-transcriptional regions in a previous study [7]. CR1 with a length of 309 bp and CR2 with a length of 307 bp shared a 263-bp identical segment (Fig. 2a). CR1 and R1 were annotated as full-length RNAs cleaved from the minor coding strand (N-strand) primary transcript, whereas CR2 and R2 were annotated as DNA regions (Table 2) covered by four transient RNAs. However, the Transcription Initiation Termination Sites (TISs) and the Transcription Termination Sites (TTSs) of the mt primary transcripts of ticks are still not determined due to insufficient data available.

**Table 2:** Precise annotations of the *Dermacentor silvarum* mt genome

Gene	Strand	Start	End	Length
tRNA-Met	(+)	1	67	67
ND2	(+)	68	1028	961
tRNA-Trp	(+)	1029	1091	63
tRNA-TyrAS	(+)	1092	1149	58
COI	(+)	1150	2686	1537
COII	(+)	2687	3359	673
tRNA-Lys	(+)	3360	3429	70
tRNA-Asp	(+)	3430	3491	62
ATP8/6	(+)	3492	4326	835
COIII	(+)	4327	5104	778
tRNA-Gly	(+)	5105	5171	67
ND3	(+)	5172	5511	340
tRNA-Ala	(+)	5512	5576	65
Intergenic	(+)	5577	5578	2
tRNA-Arg	(+)	5579	5640	62
tRNA-Asn	(+)	5641	5708	68
Intergenic	(+)	5709	5712	4
tRNA-Ser	(+)	5713	5769	57
tRNA-Glu	(+)	5770	5834	65
R2	*	5825	5987	163
HAS1	(+)	5835	9258	3424
tRNA-Ile	(+)	9259	9322	64
HAS2	(+)	9323	12,930	3608
tRNA-Thr	(+)	12,931	12,991	61
tRNA-ProAS/ND6	(+)	12,992	13,503	512
Cytb	(+)	13,504	14,585	1082
tRNA-Ser	(+)	14,586	14,652	67
tRNA-LeuAS/CR2	(+)	14,653	15,023	371
CR2	*	14,717	15,023	307
tRNA-Cys	(+)	15,024	15,086	63
Intergenic	(+)	15,087	15,094	8
tRNA-Tyr	(−)	1090	1151	62
LAS1	(−)	1152	5984	4833
ND1	(−)	5985	6903	919
tRNA-Leu	(−)	6904	6964	61
16S rRNA	(−)	6965	8185	1221
tRNA-Val	(−)	8186	8247	62
12S rRNA	(−)	8248	8949	702
CR1	(−)	8950	9258	309
tRNA-IleAS	(−)	9259	9323	65
tRNA-Gln	(−)	9324	9390	67
R1	(−)	9391	9559	169
tRNA-Phe	(−)	9560	9620	61
ND5	(−)	9621	11,275	1655
tRNA-His	(−)	11,276	11,341	66
ND4/4 L	(−)	11,342	12,928	1587
tRNA-ThrAS	(−)	12,929	12,991	63
tRNA-Pro	(−)	12,992	13,055	64
LAS2	(−)	13,056	14,654	1599
tRNA-Leu	(−)	14,655	14,716	62
LAS3	(−)	14,717	1089	1467

This reference sequence is available at the NCBI GenBank database under the accession number MN347015. J(+) and N(−) represent the major and minor coding strands of the mt genome, respectively. Control Region 1 (CR1) and tandem Repeat 1 (R1) were annotated as full-length RNAs cleaved from the minor coding strand (N-strand) primary transcript, whereas CR2 and R2 were annotated as DNA regions (*). The “AS” suffix represents antisense. H-strand Antisense Segment 1 (HAS 1) represents R2/ND1AS/tRNA^Leu^AS/(16S rRNA)AS/tRNA^Val^AS/(12S rRNA)AS/CR1. HAS2 represents tRNA^Gln^AS/R1/tRNA^Phe^AS/ND5AS/tRNA^His^AS/(ND4/4 L)AS. L-strand Antisense Segment 1 (LAS1) represents COIAS/COIIAS/tRNA^Lys^AS/tRNA-^Asp^AS/(ATP8/6)AS/COIIIAS/tRNA^Gly^AS/ND3AS/tRNA^Ala^AS/tRNA^Arg^AS/tRNA^Asn^AS/tRNA^Ser^AS/tRNA^Glu^AS/R2. LAS2 represents ND6AS/CytbAS/tRNA^Ser^AS. LAS3 represents CR2/tRNA^Cys^AS/tRNA^Met^AS/ND2AS/tRNA^Trp^AS

Using precise annotations, we obtained two new findings about the *D. silvarum* mt tRNAs. The first involved six mt tRNA genes, from which atypical tRNAs with no D-arm or an unstable T-arm were inferred [9]. One of tRNA^Ser^s(mtDNA: 5713:5769) and tRNA^Cys^ (Fig. 2b) had no D-arms, whereas tRNA^Ala^ (Fig. 2b), tRNA^Glu^, tRNA^Tyr^ and tRNA^Phe^ had unstable T-arms. Another new finding was that the intergenic regions between tick mt tRNA genes are longer than those in mammals except a novel 31-nt ncRNA [4], which was generated in the gene order rearrangement of mammalian mt tRNA genes. Although these intergenic regions in ticks were cleaved between their neighbouring tRNAs to form small RNAs (sRNAs) shorter than 10 bp, they are not likely to have biological functions, in our view. One typical example of a sRNA was A[U]_7_, between tRNA^Cys^ and tRNA^Met^ (Fig. 2b). Based on these two findings, we found that 1 × n STRs involved both intergenic regions (e.g., A[U]_7_) and atypical mt tRNAs (e.g., [A]_5_ in tRNA^Cys^). Comparison of tRNA^Ser^ and tRNA^Cys^ suggested that tRNA^Cys^ (Fig. 2b) with no D-arm had an [A]_5_ insertion that formed a large loop. Given that the tRNA^Cys^ DNA sequence had too little evolutionary conservation to allow for a STR insertion, it proved a long-standing hypothesis that atypical tRNAs do not have biological functions.

R1 and R2 (Fig. 3) were predicted to be two non-coding and non-transcriptional regions in the previous study [7]. In the present study, however, they were proven to be transcribed on two strands. The repeat units in R1 were reverse complements of those in R2 (Fig. 3b). Our DNA-seq data showed that the copy numbers of R1 and R2 exhibited great diversity within an individual, which confirmed a finding from our previous study of the *E. fullo* mt genome [5]. Since repeat units in R1 and R2 were reverse complements, we used PCR amplification (Table 1) coupled with Sanger sequencing to further investigate R1 sequences in more than 100 individual ticks from four species (*D. silvarum*, *D. nuttalli, D. marginatus* and *D. niveus*) and obtained the following results: (1) for each individual tick, the R1 sequence obtained using Sanger sequencing is actually a consensus sequence of a large number of heterogeneous sequences; (2) copy numbers were distributed between 2 and 5 for all studied repeat units, with one partial repeat unit counted as 1; (3) in total, three types of repeat units of R1 with lengths of 28, 34 and 44 bp (types 1, 2 and 3, respectively) were identified (Fig. 3b) and noted as R_28_, R_34_ and R_44_; (4) in general, R1 sequences from individual ticks of one species comprised repeat units of one type and R1 sequences from individual ticks of the same species from different places could have different copy numbers; and (5) of the four species of ticks we studied, *D. nuttalli* and *D. niveus* had a R1 which was composed of type 3 units, whereas *D. silvarum* had a R1 which was composed of type 2 or type 3 units. As for *D. marginatus*, most of the R1s were composed of the type 3 units; however, a few had R1s composed of the types 1 and 2 hybrid units, noted as [R_34_]_l_-[R_28_]_m_-[R_34_]_n_, where l, m and n represent the copy numbers. The discovery of these “hybrid” units suggested to us that mt DNA recombination may occur within an individual tick, resulting in the insertion of [R_28_]_m_ into [R_34_]_l + n_. This confirmed our proposal of DNA-recombination events in a previous study of the *E. fullo* mt genome [5]. In that previous study, the insertion of segments A and B into STR [R_87_] _l + m + n_ resulted in [R_87_]_l_-A-[R_87_]_m_-B-[R_87_]_n_.

### Discovery of a transposon-like element

In a previous study, the repeat unit was conceived as the “tick box”—a degenerate 17-bp sequence motif that may be involved in the 3′ formation of ND1 and tRNA^Glu^ transcripts in all major tick lineages [7, 10, 11]. A large translocated segment (LT1) spanning from ND1 to tRNA^Gln^ was first reported in 1998 [12, 13] and the presence of the “tick box” motif at both ends of this LT1 indicated its involvement in recombination events that are responsible for known Metastriata ticks [12–14]. Metastriata genome rearrangements have been found in all Metastriata ticks studied [8, 10–18] (Fig. 1a). In the present study, LT1 was corrected to span R2, ND1, tRNA^Leu^, 16S rRNA, tRNA^Val^, 12S rRNA, CR1, tRNA^Ile^, tRNA^Gln^ and R1 (Fig. 3a) in the reference genome using precise annotations. Given that nearly half of the human genome is various types of transposable elements that contain repetitive DNA sequences [19], we hypothesized that LT1 is a transposon, with R1 and R2 as invert repeats (IRs) and genes from ND1 to tRNA^Gln^ as insert sequences (ISs). To test our hypothesis, we sought structural variation (Methods) in the *D. silvarum* mt genome to determine the occurrence of LT1 translocation events. The results proved the occurrence of LT1 inversions within an individual tick (Fig. 3a).

Since LT1 inversions were rare, 4.1 Gbp DNA-seq data were generated to cover ~ 427,247× (4.09 Gbp/9.58 Kbp) of L1 in the *D. silvarum* mt genome to detect the LT1 inversions. As the dominant copy number was five for both R1 and R2, we used 34 × 5 STR to represent R1 and R2 in the *D. silvarum* mt genome (Fig. 3c). Thus, R1 and R2 in *D. silvarum* are ~ 170-bp long (34 × 5), which is longer than the reads in the 2 × 150 bp DNA-seq data. We had to sequence the same library using 2 × 250 bp sequencing to validate the reference genome and the LT1 inversion (Methods). The substantial diversity in R1 and R2 copy numbers within an individual tick rendered great diversity in LT1. However, we did not obtain full-length sequences of LT1 due to sequence-length limitations in the DNA-seq data. Therefore, we were unable to determine whether R1 and R2 had the same copy numbers within one LT1.

### Copy number variation of STRs in the mt genomes within an individual animal

By mapping DNA-seq data to the *D. silvarum* mt genome, variation detection (Methods) was performed to report two types of DNA variation—SNPs and small insertions/deletions (InDels). Almost all the detected DNA variation within a *D. silvarum* tick was Copy Number Variation (CNV) of STRs caused by InDels of one or more entire repeat units, whereas SNPs were not detected. We defined the STR position as the genomic position of the first nucleotide of the reference STR. For example, [G]_8_ was designated as the reference STR at position 1810, because it occurred most frequently in mtDNAs within one individual tick (Table 3); the alternative alleles of [G]_8_ included [G]_6_, [G]_7_, [G]_9_ and [G]_10_. Importantly, it was found that almost all of the STRs had multiple variants, particularly those with copy numbers greater than 5. The detection of CNV of STRs was reliable, based on the following reasons: (1) PCR amplification and deep DNA sequencing produces a high signal-to-noise ratio in the detection of DNA variation; (2) the Illumina sequencer generates very rare InDel errors, i.e. few per million bases [21]; (3) it is impossible for sequencing or alignment errors to result in 2-bp InDels in 2 × n STR (e.g., [TA]_9_); (4) the alternative allele ratios (Methods) at some positions were significantly higher and the highest ratio reached was ~ 32% at position 3441 (Table 3); (5) the same CNV of STRs caused diversity within and between individual ticks, e.g., [TA]_9_ in our genome (Table 3) and [TA]_5_ in NC_026552.1; (6) CNV of STRs was detected in other animal species, including *E. fullo* [5], mouse; and (7) Almost all the detected DNA variation within a single human cell were CNV of STRs, whereas SNPs were not detected using a colon cancer single cell RNA-seq (scRNA-seq) dataset (SRA: SRP113436).

**Table 3:** Mitochondrial CNV of STRs within a *D. silvarum* individual

Position	Gene	Ref	Allele	Depth
1810	COI	[G]_8_	Ref/+ 1G/−1G/+2GG/T/G/C/+ 1 T/*/−2GG	352,526/6177/3116/150/32/25/19/19/10/6
2573	COI	[A]_9_	Ref/+ 1A/− 1A/T/+2AA/A/+3AAA/−2AA	277,857/7636/1788/223/170/13/9/8
3441	tRNA-Asp	[A]_8_	Ref/+ 1A/+2AA/−1A/+ 1C/+3AAA/+ 1G/G/C/A	438,644/195894/3402/1011/69/58/17/10/7/5
3619	ATP8/6	[A]_10_	Ref/+ 1A/−1A/+2AA/−2AA/T/+3AAA/A/C	400,525/23182/9717/997/185/137/49/14/11
4592	COIII	[T]_11_	Ref/+ 1 T/−1 T/+2TT/−2TT/+3TTT/T/+ 1A	107,531/10859/6363/1027/140/110/20/10
5524	tRNA-Ala	[A]_10_	Ref/+1A/−1A/+2AA/−2AA/A/+3AAA	97,659/3329/2622/131/27/19/9
6361	ND1	[A]_10_	Ref/+1A/−1A/+2AA/−2AA/+3AAA/C	94,957/4984/3410/313/18/17/8
7324	16S rRNA	[TA]_9_	Ref/−2TA/+2TA	93,454/4152/3088
7737	16S rRNA	[A]_9_	Ref/+1A/−1A/+2AA/C/A	89,380/1277/890/32/6/5
8042	16S rRNA	[T]_9_	Ref/+1 T/−1 T/+2TT/−2TT/G	96,225/1795/1552/40/8/5
8243	12S/tRNA-Val	[T]_10_	Ref/+1 T/−1 T/+2TT/−2TT/+3TTT/G	99,234/3993/2326/151/31/8/7
8885	12S rRNA	[T]_10_	Ref/+1 T/−1 T/+2TT/−2TT/T/+3TTT	86,875/4767/3630/287/38/36/18
10,026	ND5	[A]_10_	Ref/−1A/+1A/+2AA/−2AA/+3AAA/C	99,166/5465/3630/134/82/6/5
10,298	ND5	[A]_13_	Ref/−1A/+1A/−2AA/+2AA/−3AAA/+3AAA/C	91,464/17434/7221/2402/721/241/61/5
11,216	ND5	[A]_17_	Ref/−1A/+1A/−2AA/+2AA/−3AAA/+3AAA/A/*/+2TA/+ 1C	138,074/26622/18595/4443/2859/542/413/96/24/5/5
11,483	ND4/4 L	[TA]_6_	Ref/−2TA/+2TA/A/T	200,829/3596/2031/19/7
11,962	ND4/4 L	[A]_11_	Ref/−1A/+1A/−2AA/+2AA/C/+3AAA/A/−3AAA	203,481/18678/10550/646/496/28/20/11/10
12,082	ND4/4 L	[A]_8_	Ref/−1A/+1A/T/+2AA/A	215,342/1501/821/48/12/7
12,505	ND4/4 L	[A]_9_	Ref/−1A/+1A/+2AA/C/−2AA	227,869/3067/2631/47/20/9
12,697	ND4/4 L	[A]_8_	Ref/+1A/−1A/+2AA/C/A/G	230,254/1922/497/23/22/6/6

This reference sequence (GenBank: MN347015) uses the consensus DNA sequence of one *Dermacentor silvarum* individual collected from Qiangyang, Gansu province of China. All the STR variants were selected using a very strict parameter (Methods). Ref was designated as the STR reference, as it occured the most frequently in mtDNAs within one individual tick. In the allele column, insertions and deletions were noted as “+” and “-” following the specifications in pileup format [20]. The numbers in the depth column are one-to-one corresponding to the variants in the allele column. For example, the STR position 1810 had a reference sequence [G]_8_ and a variant [G]_9_ noted by “+G”, which had a depth 6177

Using a very strict parameter (Methods), we selected 20 STR positions in the *D. silvarum* mt genome for further study (Table 3). STRs at 18 positions were 1 × n STR and those at the other 2 positions were 2 × n STR. Almost all of the STRs were composed of A or T, except [G]_8_ at position 1810. All of the reference STRs and their variants had copy numbers greater than 5. Among all of the variants, 1 × n STR occurred much more frequently than m × n STR (m > 1). Thirteen STR positions in the protein-coding genes had identical sequences between individuals, exhibiting a high degree of evolutionary conservation; however, the other seven STR positions in the tRNA and rRNA genes exhibited variation. For example, [A]_10_, [TA]_9_, [T]_9_ and [T]_10_ at positions 5524, 7324, 8042 and 8243 in our mt genome changed to [A]_8_, [TA]_5_, [T]_8_ and [T]_6_ at positions 5524, 7324, 8042 and 8243 in NC_026552.1 (Table 3). The CNV of STRs in the protein-coding genes resulted in frameshift mutations in the proteins, which may be deleterious [22]. For example, the COI gene has a 1524-bp Coding Sequence (CDS) for a 308-aa protein. The variant [G]_9_ at genomic position 1810 (Table 3) results in a 765-bp CDS for a 255-aa truncated COI protein. This finding inspired us to investigate if animal cells have mechanisms to remove mitochondria containing deleterious mutations or inhibit the expression of the deleterious variants, as they can cause loss of function or diseases. We did not, however, obtain deep RNA-seq data for *D. silvarum*. We had to compare the STR variants in the *E. fullo* mt genome [5] at the DNA level and RNA levels, using the DNA-seq and RNA-seq data (SRA: SRP174926). However, we found no significant differences between them. This suggested that deleterious STR mutations can irreversibly change the proteins made from their mRNAs and that mitochondria containing deleterious mutations may accumulate in cells.

## Discussion

SNPs, accepted as the most common type of genetic variation, play a dominant role in studies in almost all biological fields; however, research of STRs has been limited to specialized fields. In particular, the use of SNPs is becoming dominant in the studies of mitochondria, e.g., mt heterogeneity. After further analysis of tick, insect, mouse and human DNA-seq and RNA-seq data, we found that a large number of STR variants had been missed in variation detection, as most research has focused only on the detection of SNPs using WGS data. Although SNP platforms are higher throughput and more cost-effective for genome scans, STRs remain highly informative measures of DNA variation for linkage and association studies. Using PCR coupled with very deep DNA-seq data, we were able to allow three gaps in genome alignment and still ensure the detection accuracy of STRs. Based on these results, we proved that WGS and RNA-seq data can also be used to detect CNV of STRs with careful adjustment of parameters (Methods). Future studies are necessary on the origins, mutation rates and effects of CNV of STRs in, but not limited to, animal mt genomes.

Both 1 × n STRs and 34 × n STRs had the same variation pattern; 34 × n STRs can be produced by DNA recombination, however, the cause of 1 × n STRs remains unclear. One possible cause of 1 × n STRs is replication slippage [23]. According to the “replication slippage” theory, DNA polymerase causes mismatches between DNA strands while being replicated during meiosis. The DNA polymerase can then slip while moving along the template strand and continue on the wrong nucleotide. Another cause might be point mutations, based on a study comparing human and primate genomes [24]. Both replication slippage and point mutations can not be used to explain the occurrence of m × n STRs (m = 2, 3, 4, 5…). As both 1 × n STRs and 34 × n STRs had the same variation pattern, they are probable to have the same causes.

Unique DNA sequences in a genome have a very low variation/mutation rate (approximately 10^− 9^ nt per generation), whereas the mutation rates in STRs are several orders of magnitude higher, ranging from 10^− 6^ to 10^− 2^ nt per generation for each locus [25]. Direct estimates of STR mutation rates have been made in numerous organisms from insects to humans, e.g., *Schistocerca gregaria* 2.1 × 10^− 4^ [26]. However, most research focuses on SNPs, rather than CNV of STRs in mt genomes. In the present study, we only detected CNV of STRs in mt genomes within an individual animal and found that the alternative allele ratio was distributed from less than 0.01% to ~ 33%. This suggested that CNV of STRs occurrences varied significantly along the genomes such that mutations concentrated at certain positions (e.g., 20 STR positions for the *D. silvarum* mt genome). Future studies must be performed to estimate STR mutation rates of mitochondria to test if they have correlations with life expectancy of different animals, using individuals at different developmental stages.

It is well accepted that many STRs are located in non-coding DNA and are biologically silent, while others are located in regulatory or even coding DNA. STRs located in regulatory, intron and transposon regions are beyond the scope of the present study. However, our studies showed there were no significant differences between the alternative allele ratios of STR positions in protein-coding gene regions and those in tRNA and rRNA gene regions in tick mt genomes. Particularly, mitochondria containing deleterious mutations can accumulate in cells and deleterious STR mutations irreversibly change the proteins made from their mRNAs. This suggested that deleterious STR mutations in mitochondria cause aging and diseases, also known as Tandem Repeat Disorders (TRDs). Huntington’s disease, as one of the famous TRDs, occurs in the context of expanded glutamine [CAG]_n_ repeats. Several other human diseases have also been linked to CNV of mt STRs. One famous example—breast cancer (BC)—has been linked to D_310_ variation [27]. The telomeres at the ends of the chromosomes consist of [TTAGGG]_n_ in vertebrates. Thus, both telomeres and mitochondria are linked to ageing/senescence by CNV of STRs.

## Conclusion

In the present study, we used long-PCR amplification coupled with NGS to obtain complete mt genomes of individual ticks and performed precise annotation of these genomes. The discovery of a transposon-like element shed light on the mechanisms of mt gene order rearrangement and genomic structural variation, especially with additional data from more tick species. The second finding may pave the way to an eventual understanding of the mechanisms of mt DNA variation. The comparison between interindividual and intraindividual variation showed that STRs with shorter repeat units (e.g., 1 × n STRs) and STRs with longer repeat units (e.g., 34 × n STRs) had the same variation pattern. This finding will encourage reconsideration of the importance of STRs as well as a unified study of CNV of STRs with shorter and longer repeat units in both nuclear and mt genomes.

## Methods

Individual ticks from four species (*D. silvarum*, *D. nuttalli, D. marginatus* and *D. niveus*) of the genus *Dermacentor* were collected from different places in China and were identified using a stereoscopic microscope according to [28]. Total DNA was isolated from individual ticks using QIAamp DNA Mini Kit (Qiagen, Germany), following its protocol. Long-PCR amplification of each entire mt genome in L1, L2, L3 and L4 (Fig. 1a) was performed using Long PCR Mix (Sino-Novel, China) and the PCR reaction mixture was incubated at 95 °C for 3 min, followed by 34 PCR cycles (30 s at 98 °C, 30s at 55 °C and 5 min at 68 °C for each cycle) with the final 5 min extension at 72 °C, while PCR amplification of short segments (CR1, CR2, R1 and R2) was performed using Taq PCR Mix (Sino-Novel, China) and the PCR reaction mixture was incubated at 95 °C for 3 min, followed by 34 PCR cycles (30 s at 95 °C, 30s at 55 °C and 1 min at 72 °C for each cycle) with the final 5 min extension at 72 °C. All the primers and PCR reaction conditions are listed in Table 1. The PCR-amplified L1, L2, L3 and L4 of *D. silvarum* and L3 and L4 of *D. nuttalli* and *D. marginatus* were used to construct ~ 250-bp-size libraries, respectively and sequenced using 2 × 150 bp paired-end strategy on the Illumina HiSeq X Ten platform. The PCR-amplified L1 of *D. silvarum* was also used to construct one ~ 350-bp-size library and sequenced using 2 × 250 bp paired-end strategy on the Illumina HiSeq 2500 platform. On average, 2 Gbp DNA-seq data was obtained for L1, L2, L3 and L4, respectively, thus, 4 Gbp data was obtained for each mt genome. In total, 10 runs of DNA-seq data (L1 of 2 × 250, L1, L2, L3 and L4) of *D. silvarum* were submitted to the NCBI SRA database under the project accession number SRP178347. The assembly of tick mt genomes was performed using the software MIRA 4.0.2. The complete mt genome sequence of *D. silvarum* is available at the NCBI GenBank database under the accession number MN347015. However, precise annotations of the *D. silvarum* mt genome need be seen in Table 2, as the NCBI GenBank database is not able to accept this new annotation format. The PCR-amplified short segments (Table 1) were sequenced using Sanger sequencing. Using the software BWA v0.5.7, DNA-seq reads were aligned to the *D. silvarum* mt genome with parameters (aln -n 3 -o 1 -e 2 -l 15 -k 1) to detect DNA variation. CNV of STRs (Table 3) was detected by single-end alignment using the 2 × 150 bp DNA-seq data of L1 and L2, and then, validated using the 2 × 150 bp DNA-seq data of L3 and L4 and 2 × 250 bp DNA-seq data of L1. The alternative allele ratio was calculated by the depth of all alternative alleles divided by the total depth on the genomic position. The 20 STR positions were selected using a strict parameter, which required the alternative allele ratio above 1%.

Total RNA was isolated from ticks to construct sRNA-seq libraries, following the protocol [29] and these libraries were sequenced using 50-bp single-end strategy on the Illumina HiSeq 2500 platform. In total, two, two and two runs of sRNA-seq data from *D. silvarum*, *D. nuttalli* and *D. marginatus* were submitted to the NCBI SRA database under the project accession number SRP178347. Total RNA was isolated from *D. silvarum* to construct one PacBio full-length cDNA library, following the protocol [2] and this library was sequenced on the PacBio Sequel sequencer to obtain the PacBio full-length transcriptome data. The cleaning and quality control of sRNA-seq and the PacBio full-length transcriptome data were performed using the pipeline Fastq_clean [30]. Using the software bowtie v0.12.7, sRNA-seq reads were aligned to the *D. silvarum* mt genome with one mismatch for precise annotation. To confirm the precise annotation of the *D. silvarum* mt genome, the PacBio full-length transcriptome data was used to follow the same procedure as [5]. The structural variation detection was performed following the protocol as [31]. Statistics computing and graphics were conducted using the software R v2.15.3 the Bioconductor packages [32].

## Data Availability

The complete mitochondrial genome sequence of *Dermacentor silvarum* is available at the NCBI GenBank database under the accession number MN347015 and the raw data is available at the NCBI SRA database under the accession number SRP178347.

## Abbreviations

NGS: Next-Generation Sequencing;
mt: mitochondrial
CNV: Copy Number Variation
STR: Short Tandem Repeat
SNP: Single Nucleotide Polymorphism
NCBI: National Center for Biotechnology Information
SRA: Sequence Read Archive
lncRNAs: long non-coding RNAs
sRNA: small RNA
nt: nucleotide
ncRNA: Non-coding RNA
CSB: Conserved Sequence Block
WGS: Whole-Genome Sequencing
NUMT: Nuclear Mitochondrial DNA
CR: Control Region
J-strand: major coding strand
DNA-seq: DNA sequencing
SSR: Simple Sequence Repeat
R: tandem repeat
sRNA-seq: small RNA sequencing
N-strand: minor coding strand
LT: large translocated segment
IR: invert repeat
IS: insert sequence
InDel: insertion/deletion
scRNA-seq: single cell RNA-seq
CDS: Coding Sequence
TRD: Tandem Repeat Disorder
BC: breast cancer
